# Gender differences in the prevalence and impact factors of adolescent dissociative symptoms during the coronavirus disease 2019 pandemic

**DOI:** 10.1038/s41598-022-24750-0

**Published:** 2022-11-23

**Authors:** Qinglin Cheng, Gang Zhao, Junfang Chen, Yuanyuan Deng, Li Xie, Le Wang

**Affiliations:** 1 Division of Infectious Diseases, Hangzhou Center for Disease Control and Prevention, 568 Mingshi Road, Hangzhou, 310021 China; 2 School of Public Health, Hangzhou Normal University, Hangzhou, 310021 China

**Keywords:** Human behaviour, Risk factors

## Abstract

The purpose of this study was to explore the differences between the prevalence and impact factors of adolescent dissociative symptoms (ADSs) by using sex-stratification during the coronavirus disease 2019 (COVID-19) pandemic. A school-based, two-center cross-sectional study was conducted in Hangzhou City, China, between January 1, 2021 and April 30, 2022. The sample included 1,916 adolescents aged 13–18 years that were randomly selected using a multiphase, stratified, cluster sampling technique. A two-stage assessment procedure was used to find out the ADSs. We used a multivariate logistic regression analysis to assess the impact factors of ADSs during the COVID-19 pandemic. The adolescent dissociative scores (*t* = 4.88, *P* < 0.001) and positive ADSs rate (Chi-square = 15.76, *P* < 0.001) in males were higher than in females. Gender-stratified, stepwise multiple logistic regression analysis revealed that the conflict relationship of teacher-student [adjusted odds ratio (AOR) 1.06, 95% confidence interval (CI) 1.01–1.10], family expressiveness (AOR 0.87, 95% CI 0.78–0.98), family conflict (AOR 1.15, 95% CI 1.05–1.27), family organization (AOR 0.88, 95% CI 0.78–0.99), and family cohesion (AOR 0.87, 95% CI 0.77–0.99) were linked to ADSs only in males, while individual psychological states of somatic complaint (AOR 1.04, 95% CI 1.00–1.08) and paranoid ideation (AOR 1.09, 95% CI 1.01–1.19) were associated with female ADSs only. The ADSs seemed to be prevalent in Hangzhou City, studied during the COVID-19 pandemic. Gender differences in the prevalence and impact factors of dissociative symptoms seem to be significant among adolescents. Thus, gender-specific intervention programs against ADSs should be considered as reducing this risk.

## Introduction

Adolescence is a critical period of mental development and greater vulnerability that involves many profound biopsychosocial transformations^1,2^. Unlike infection control measures from other countries, China imposed the 'dynamic zero-COVID policy' measure to reduce the coronavirus disease 2019 (COVID-19) transmission as of now. Thus, coping with the COVID-19 pandemic and complying with restrictive measures might be easier to produce psychological harms in adolescents than in other age groups^3^. Recent evidence from several countries (i.e., Canada, China, the United Kingdom, Turkey, and USA) indicated that the COVID-19 pandemic caused negative impacts on adolescents' mental health^3–7^. The COVID-19 pandemic has a tremendous impact on adolescents' mental development, as described above. Recent research suggested that traumatic stress reactions (TSRs) during the COVID-19 pandemic are prevalent^8,9^. Notably, the trauma-induced dissociative symptoms (DSs) of non-integration were prone to occur in traumatized children and adolescents^10–12^. Data from several studies also found that there was a high proportion of patients among adolescents' psychiatry with significant DSs after early childhood traumatization^10,13–15^. Neurobiological studies indicated that active, early and effective identification and treatment interventions on early stressful adolescent experiences were very important for the prevention and treatment of adolescent dissociative disorder (ADD)^16^. Hence, DSs is an early sign before the onset of ADD in children and adolescents.

In recent studies, there was increasing recognition that the mental harm reduction of COVID-19 pandemic caused the consideration of potential impact factors^17^. There is an urgent need for scientifically proven and improved potentially modifiable impact factors associated with ADD during the COVID-19 pandemic. Some studies suggested that a modifiable impact factors associated with ADD, such as DSs, should be raised^18,19^. According to the Diagnostic and Statistical Manual of Mental Disorders 5th edition (DSM-5), DSs refer to abnormal integration of thoughts, feelings, and experiences (e.g., disengagement, memory disturbances, derealization, and identity alteration) in the stream of consciousness and memory^20^. DSs result from preexisting personality traits that are defined by high DSs scores^21^. As DSs research accumulates, researchers have reported a strong association between a history of sexual abuse and adolescent DSs (ADSs)^22^. ADSs are more prevalent in girls than in boys. ADSs tend to worsen with increasing age and become more similar to adult presentations^10^. Extensive research has shown that ADSs may precede the onset of the disorder and could be a risk factor for the development of bipolar disorder^23^. ADSs are frequently associated with a worse prognosis and more frequent mood swings^24^. Although professional interest in ADSs has increased in the past two decades, the assessment and intervention of ADSs is still relatively unfamiliar to clinicians or researchers.

Previous studies have suggested that ADSs could induce several adverse outcomes, including ADD, non-suicidal self-injury, alcohol use disorder, and antisocial personality disorder^10,19–24^. These studies support a link between DSs and adverse outcomes, and provide evidence for the effects of the early signs of dissociative disorder (DD) overtime. But we still found little reports attributed to the mediation effects of ADSs. For example, though the relevance between DSs and adverse outcomes has been well reported^10,18^, no specific prevalence nor risk estimates are available pertaining to ADSs. Previous studies over the last decade have provided important information on the treatment of DSs^25,26^. Such approaches, however, have failed to address the evaluation and intervention of ADSs. According to our review of the literature, little research has been performed based on the gender-specific risk assessment of ADSs^24,25,27–29^. So far, there are few studies that have investigated gender characteristics in the prevalence and impact factors of ADSs during the COVID-19 pandemic. Early identification and effective intervention may reduce the impact of ADSs and prevent the occurrence of several adverse outcomes and the persistence of DD into adulthood^19,24,27–29^. This suggested that we might first attempt to identify those adolescents who were at risk of developing DSs at an early stage (e.g., age 13–18 years). Given the studies published to date, developmental patterns of the gender difference in ADSs have been largely neglected^12,15,16,18,19,27–29^. Of particular concern are unclear pertaining to gender characteristics in ADSs during the COVID-19 pandemic. In order to make up the above mentioned deficiencies, the present study will examine the gender characteristics in ADSs from dissociative scores, the prevalence, and impact factors (i.e., social-demographic, social, family, school, and psychological factors) during the COVID-19.

To effectively reduce ADSs risk and prevalence rates, the potential impact factors of sex-specific ADSs should be investigated using large-scale community and epidemiologic studies during the COVID-19 pandemic, further developing effective interventions against ADD and other mental disorders. We assumed that, if it was possible to determine the environmental variables that were related to ADSs, environmental factors might mediate the association between gender and ADSs. Thus, we performed a two-center, cross-sectional study to discuss gender differences based on the prevalence and impact factors of ADSs across Hangzhou City during the COVID-19 pandemic.

## Materials and methods

### Sample size calculation

To calculate the sample size, we used the following formula^30^:

$$N = \frac{{Z^{2} \times \left( {1 - P} \right)P}}{{\delta^{2} }} \times deff$$

where *N* = sample size; Z = Z statistic for confidence level; *P* = expected prevalence rate; $\delta$= allowable error. We used the design effect *deff* to calculate the sample size due to multistage sampling methods in the present study.

In this study, the sample size was calculated to meet the following conditions: (1) the preliminary estimate of ADSs prevalence was 23.6% in the pre‐survey study; (2) the allowable error was taken to ensure accuracy by using 3%; (3) for a 95% confidence level, which was conventional, the Z-value was 1.96; (4) the investigators present their results with 95% confidence intervals (CI) and *deff* = 2. In this study, the sample size was calculated as 1,539. The calculation of sample size needed to consider students who were lost in the follow-up survey, student rejection rate, sampling error, and stratification factors. The final sample size was satisfied in our study as 2,160.

### Study methods

**Figure 1:**
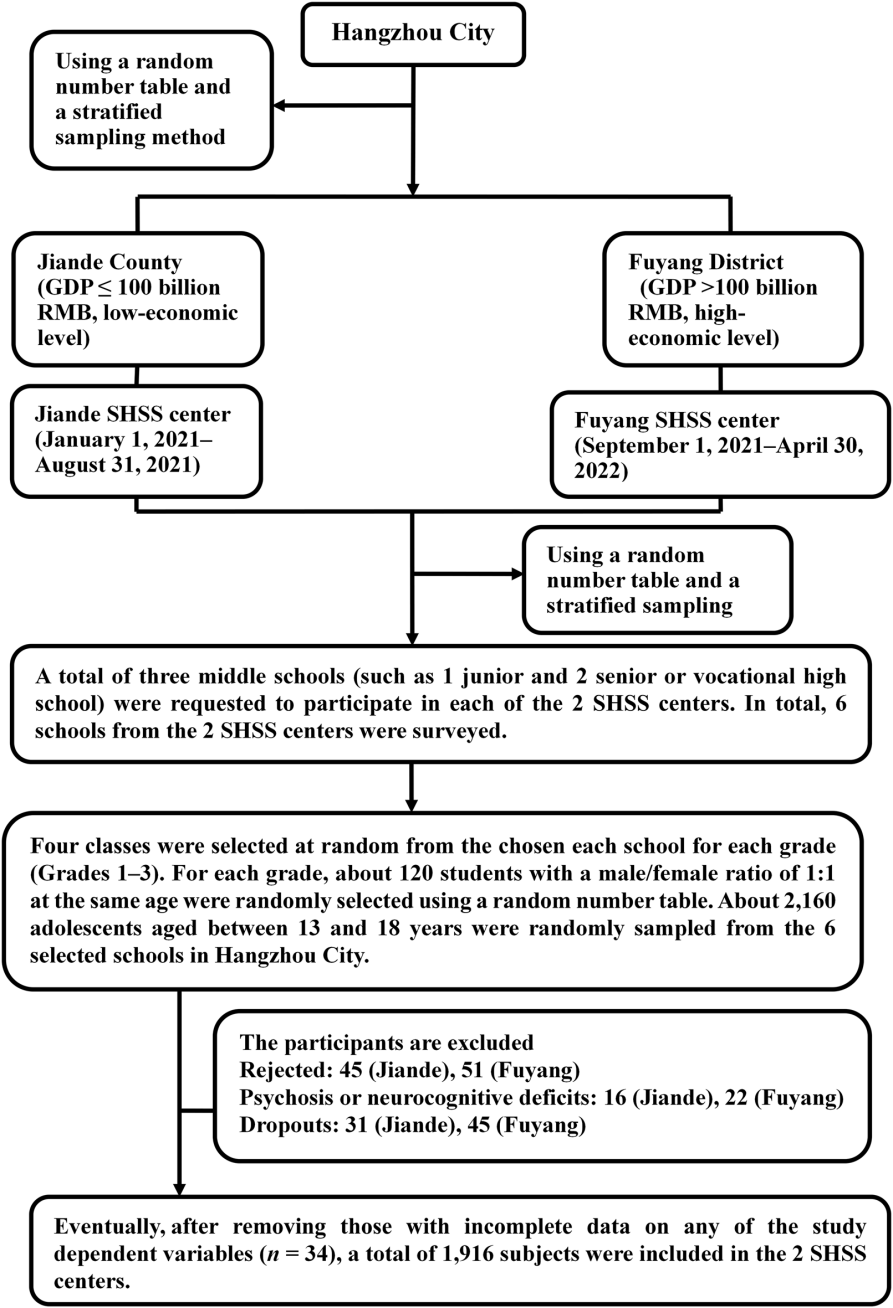
Workflow in this study. *GDP* gross domestic product, *SHSS* School Health Surveillance System.

This study was a school-based, cross-sectional study, in which six schools were recruited in Hangzhou City from January 1, 2021 to April 30, 2022. We used a random number table, and a stratified cluster sampling method to select participants. A sample of 360 students aged 13–18 years at each of the enrolled schools were randomly selected from two School Health Surveillance System (SHSS) centers (including Jiande County Center and Fuyang District Center in Hangzhou City). To be included, participants had to be adolescents aged 13–18 years, and could converse in Chinese. Participants were excluded if they had a history of psychosis or neurocognitive deficits or received a secondary mental health service. Figure 1 provides further details of this study.

In the present study, ADSs was estimated at a two-stage appraisal procedure. In the first stage, ADSs were screened by the Adolescent Dissociative Experience Scale (A-DES)^31^. In the second stage, subjects whose responses to the A-DES suggested they might have ADSs were further evaluated by three psychiatrists to get a final diagnosis. The investigators included uniformly trained psychiatrists, medical students, and school health care personnel. About 60 trained investigators performed 2 questionnaires to participants that were estimated as having ADSs. The investigators administered face-to-face evaluations and investigations in each school. In addition, 3 trained investigators were present in each school in order to guarantee the quality of our research process.

### Measures and procedures

#### ADSs measurement

We used a two-stage identification procedure to appraise ADSs. In the first stage, ADSs were screened for using the A-DES, which contained 30 items^31^. Items in the A-DES included experiences of dissociative amnesia (items 2, 5, 8, 12, 15, 22, 27), depersonalization/derealization (items 3, 6, 9, 11, 13, 17, 20, 21, 25, 26, 29, 30), absorption/imaginative involvement (items 1, 7, 10, 18, 24, 28), and passive influence (items 4, 14, 16, 19,23). The items are rated by the adolescent on an 11-point Likert scale ranging from 0 = 'never' to 10 = 'always', with no midpoint scores^31^. We used the mean of all item scores to calculate the total A-DES score (score range, 1–10 points) for the original instrument, with higher scores showing greater severity^31^. The present study used a mean score of 3 or above on the A-DES to signify pathological dissociation^32^.

Participants whose responses to the A-DES suggested that they might have ADSs (i.e. a medium or high dissociation scores ≥ 3)^32^, which were further estimated by three psychiatrists (such as three chief physicians) to get a final diagnosis as follows. First, they carefully reviewed the diagnostic criteria of ADD and related studies on DSs due to ensuring the diagnostic consistency and accuracy of the three psychiatrists. Next, the psychiatrists used the Structured Clinical Interview for DSM-5- Dissociative Disorders (SCID-D) and the Chinese Classification of Mental Disorders to diagnose all patients with ADSs, DD and other mental or personality disorders. Then, to ensure the appraisal consistency of the different psychiatrists, they used a mutual evaluation form; thus, ADSs were only confirmed when DSs were consistently diagnosed by all three psychiatrists.

During this assessment, psychiatrists took primary responsibility for the diagnosis and differential diagnosis of ADSs. The ADSs was considered if a subject had a) a medium or high dissociation scores (≥ 3); b) the exclusion of organic diseases, or other mental or personality disorders; c) no history of neurological/psychiatric illness; and d) no intelligence deficit (i.e. an intelligence quotient > 70)^33^.

The reliability of the questionnaire was tested in the pre-survey study. The responses were analyzed using Cronbach's alpha. Cronbach's alpha for all 30 items was 0.97 in the current study.

#### Social adjustment status measurement

We used the Chinese Adaptation Scale for Adolescents (CASA) to assess the social adjustment status of adolescents^34,35^. The CASA comprises three factors: emotional adaptation (6 items), social adaptation (6 items), and study and life adaptation (5 items)^35^. This CASA was a 4-point Likert scale for a total score of 17–68 points. The answer response format is a 1–4 scale, anchored at the ends with 1 = 'not at all' and 4 = 'exactly'. Higher scores indicate better social adjustment abilities in the CASA. Previous studies from our group showed that the CASA has high reliability and validity, with a Cronbach coefficient of 0.88^34^. The internal consistency was 0.90 in the present sample.

#### School environment status measurement

#### Teacher-student relationship

We used the Student–Teacher Relationship Scale (STRS) to evaluate the relationship between teachers and students^36^. The STRS is a 28-item self-report instrument, which includes four domains: intimacy, conflict, support, and satisfaction^36^. The STRS rates the extent to which they agreed with each statement using a 5-point Likert scale (1 = 'Definitely does not apply'; 2 = 'Does not really apply'; 3 = 'Neutral, not sure'; 4 = 'Applies somewhat'; and 5 = 'Definitely applies'). This STRS uses a total score of 28–140 points to assess the student–teacher relationship. The three dimensions of intimacy, support and satisfaction for the STRS reflect a positive teacher-student relationship, which higher scores indicate better teacher-student relationship. The dimension of conflict reflects a negative teacher-student relationship, in which higher scores indicate worse teacher-student relationship in the STRS. Our previous study suggested that the Chinese version of the STRS (STRS-CV) has good internal consistency and acceptable test–retest reliability (with a Cronbach coefficient of 0.87)^34^. The internal consistency was 0.83 in the present sample.

#### Peer relationship

We used the Chinese version of the Peer Relationship Inventory (CPRI) to estimate adolescents' peer relationships^37^. The CPRI is a 20-item self-report instrument, which measures three analytically derived dimensions of peer relations: social maturity, aggression, and independence^37^. The participants were asked to rate on a 5-point Likert scale (1 = 'Not at all matches'; 2 = 'Mild match'; 3 = 'Moderate match'; 4 = 'Most matches'; and 5 = 'Perfect match'). The peer relationship was estimated by using a total CPRI score (score range, 20–100 points) for the original scale, with higher scores showing better peer relationship^37^. In our prior studies, the scale has high reliability and validity, with a Cronbach coefficient of 0.90^34^. The internal consistency was 0.94 in the present sample.

#### Family environment status measurement

We used the Chinese Version of Family Environment Scale (FES-CV) to assess relationships, personal growth, and system maintenance in one's family environment^38^. The FES-CV is a 90-item self-report instrument^38^, which includes three dimensions measured by 10 subscales that include relationship dimension [cohesion (score range, 0–9 points), expressiveness (score range, 0–9 points), and conflict (score range, 0–9 points) subscales], personal growth dimension [independence (score range, 0–9 points), achievement orientation (score range, 0–9 points), intellectual-cultural orientation (score range, 0–9 points), active-recreational orientation (score range, 0–9 points), and moral-religious emphasis (score range, 0–10 points) subscales], and system maintenance dimensions [organization (score range, 0–8 points) and control (score range, 0–9 points) subscales]. The adolescents were asked to rate on dual-choice (1 = 'yes' and 0 = 'no') questionnaire with ten scales that assessed different characteristics of families. Higher scores in the subscales of cohesion, expressiveness, independence, achievement orientation, intellectual-cultural orientation, active-recreational orientation, moral-religious emphasis, and organization indicated better family relationship. Higher scores in the subscales of conflict and control signified worse family relationship. Prior studies indicated that ten subscales of the FES-CV showed moderate to excellent internal consistency (ranging from 0.63–0.75) and acceptable test–retest reliability at 0.55–0.92^34,38^. The internal consistency of this scale was 0.89 in the present sample.

#### Mental health status measurement

We used the Chinese version of Symptom Check List-90 (SCL-90-CV) to estimate the mental health status of adolescents^39^. The SCL-90-CV is a 90-item self-report instrument, which includes ten subscales (i.e., objective-comprehensive, somatic complaint, interpersonal sensitivity, depression, anxiety, hostility, phobic anxiety, paranoid ideation, psychoticism, and diet/sleeping) that respond to an individual's specific aspect of the symptomatic situation^39^. The total score of SCL-90-CV ranges from 90 to 450. Each item on the scale is measured on a 5-point Likert scale, with 1–5 representing the severity of the symptoms as follows: (1 = 'Not at all'; 2 = 'A little bit'; 3 = 'Moderately'; 4 = 'Quite a bit'; and 5 = 'Extremely'. The items relevant to each subscale are averaged to give a subscale score (score range, 1–5 points). Based on the scores of each subscale, it is possible to initially determine which subscales represent problematic psychological symptoms according to their experience in recent weeks^39^. Thus, a higher score reflected more serious psychological symptoms. Related research demonstrated that the SCL-90-CV had good internal consistency and acceptable reliability^40^. In the present sample, the internal consistency was 0.94.

### Statistical analysis

All data was double entered and verified by using EpiData 3.1 software. The analyses of all data were carried out with R (R Core Team (2021) version 4.1.2. R: A language and environment for statistical computing. R Foundation for Statistical Computing, Beijing, China; https://www.R-project.org), with the aid of the 'forestmodel' package. Markov chain Monte Carlo (MCMC) method of multiple imputation (MI) was used to handle missing data^41^. We used a MCMC approach to perform 200 imputations of each variable based on the MI procedure in R software (‘mice’ package). We evaluated the initial estimates for MCMC through the expectation–maximization algorithm.

We used the Kolmogorov–Smirnov test to assess the normality of quantitative variables. We calculated the frequencies and percentages of categorical variables. Continuous variables were presented as the mean ± standard deviation (SD).

Univariate analyses were used to separately analyze all the variables that were potentially associated with ADSs. We included in multiple regression models at a significance level of 0.10. The independent *t*-test was used to analyze continuous variables with a normal distribution. Pearson's chi-squared test was used for categorical variables.

We used multivariate logistic regression models to evaluate whether the variables, including demographics, environmental factors, and psychological factors, were associated with male or female ADSs. A stepwise procedure was used to select further the variables related to ADSs at a significance level of *P* > 0.05 for removal and a significance level of *P* ≤ 0.05 for reentry. We performed the hypothesis testing to indicate statistical significance by using a two-sided test with an alpha value of 0.05.

### Ethics approval and consent to participate

The studies involving human participants were reviewed and approved by the ethics committee of Hangzhou Center for Disease Control and Prevention. Written informed consent to participate in this study was provided by the participants or their legal guardian/next of kin. In addition, all methods were carried out in accordance with relevant guidelines and regulations.

## Results

### Baseline characteristics of the subjects

The response rate was 88.70% (1,916/2,160) in the cross-sectional study. In total, 1,916 subjects completed this survey, and 96 subjects refused to take part in the study, representing a refusal rate of 4.44% (96/2,160) at six schools in Hangzhou City. In addition, we excluded 38 participants because of psychosis or neurocognitive deficits and 76 participants because of dropouts. After removing questionnaires with incomplete data on any of the study dependent variables (*n* = 34, accounting for 1.57%), 1,916 subjects were included in subsequent analyses. Among 1,916 subjects, 924 (48.23%) were males and 992 (51.87%) were females. The average age of participants was 15.52 years (SD 1.67). Among them, the participants were 1,320 (68.89%) in rural areas, and 596 (31.11%) in urban areas.

### Differences in terms of the adolescent dissociative scores and prevalence of ADSs

The overall positive ADSs rate was 23.12% (95% CI 21.25–25.08%) (443/1,916) at 18.45% (95% CI 16.03–20.86%) (183/992) for females and 28.14% (25.26–31.16%) (260/924) for males in Hangzhou City. The adolescent dissociative scores (*t* = 4.88, *P* < 0.001) and positive ADSs rate (Chi-square = 15.76, *P* < 0.001) in males were higher than in females. The adolescents from rural areas had higher adolescent dissociative scores (*t* = 2.47, *P* = 0.014) compared to adolescents from urban areas (Table 1). The prevalence rate of male ADSs was higher in rural areas than in urban areas (Chi-square = 4.13, *P* = 0.042). However, there was no significant difference in the prevalence of female ADSs between rural and urban areas (*P* > 0.05). We also found that age did not significantly relate to the adolescent dissociative score or positive ADSs rate in adolescents (*P* > 0.05) (Table 1).

**Table 1:** Baseline characteristics of subjects (*N* = 1,916).

Variables	Total	ADSs	*Deff*	*χ2 (Phi coefficient)*	*P*-value*	Dissociative scores	*Deff*	*t* (*Cohen's d-statistic*)	*P*-value*
**Gender**									
Male	924	260 (58.69)	1	**15.76 (−0.08)**	** < 0.001**	2.77 ± 2.36	1,914	**4.88 (0.20)**	** < 0.001**
Female	992	183 (41.31)				2.29 ± 1.91			
**Age (years)**									
13–15	936	204 (46.05)	1	1.13 (0.02)	0.288	2.51 ± 2.19	1,914	−0.22 (0.10)	0.823
16–18	980	239 (53.95)				2.53 ± 2.12			
**Residence area**									
Rural	1,320	322 (72.69)	1	2.45 (−0.03)	0.118	2.60 ± 2.17	1,914	**2.47 (0.11)**	**0.014**
Urban	596	121 (27.31)				2.34 ± 2.11			

Data are presented as No. (%) or mean ± standard deviation.

*ADSs* Adolescent dissociative symptoms.

*Data in bold are statistically significant (*P* < 0.10).

### Gender differences in terms of the impact factors of ADSs

Table 2 summarizes the results of the univariate analyses of the association between potential covariates and ADSs. About 23 of the 31 tested covariates were significantly associated with male ADSs (*P* < 0.10). The significant covariates were (a) demographic characteristics, including residence area; (b) environment impact factors including social adjustment status (i.e., emotional adaptation, social adaptation, and study and life adaptation), school environment factors (i.e., the conflict relationship of teacher-student and interpersonal harmony relationship of peer), and family environment factors (i.e., expressiveness, conflict, intellectual-cultural orientation, active-recreational orientation, moral-religious emphasis, organization, and cohesion); (c) psychological factors, including 10 factors scores of SCL-90. The remaining 8 covariates, including age, age group, the positive relationship of teacher-student, the social emotion relationship of peer, the interpersonal interaction relationship of peer, family independence, family achievement orientation, and family control), did not significantly relate to male ADSs risk (*P* ≥ 0.10) (Table 2). Whilst 25 of the 31 tested covariates were significantly associated with female ADSs (*P* < 0.10). The significant covariates were (a) demographic characteristics (i.e., age); (b) environment impact factors including social adjustment status (i.e., emotional adaptation, social adaptation, and study and life adaptation), school environment factors (i.e., the positive and conflict relationships of teacher-student, and peer relationships with social emotion, interpersonal interaction, and interpersonal harmony), and family environment factors (i.e., expressiveness, conflict, intellectual-cultural orientation, moral-religious emphasis, organization, and cohesion); (c) psychological factors, including 10 factors scores of SCL-90. The remaining 6 covariates (including age, residence area, family independence, family achievement orientation, family active-recreational orientation, and family control) did not significantly relate to female ADSs risk (*P* ≥ 0.10) (Table 2).

**Table 2:** Univariate logistic regression analyses were stratified for the association between impact factors and ADSs by gender during the coronavirus disease 2019 pandemic (*N* = 1,916).

Variables	Males (*n* = 924)*		*P-*value	Females (*n* = 992)*		*P*-value
	No	Adjusted OR (95% CI)		No	Adjusted OR (95% CI)	
Sociodemoragphic characteristics						
Age (years)	924	1.07 (0.98–1.16)	0.154	992	**1.09 (0.99–1.20)**	**0.080**
Age group (years)						
16–18 (Senior high school students)	471	1.20 (0.90–1.60)	0.216	509	1.12 (0.81–1.54)	0.502
13–15 (Junior high school students)	453	Reference		483	Reference	
Residence area						
Rural	625	**1.55 (1.12–2.13)**	**0.008**	695	1.03 (0.72–1.46)	0.888
Urban	299	Reference		297	Reference	
*Environment factors*						
Social adjustment status						
Emotional adaptation	924	**0.90 (0.87–0.94)**	** < 0.001**	992	**0.86 (0.82–0.91)**	** < 0.001**
Social adaptation	924	**0.92 (0.89–0.96)**	** < 0.001**	992	**0.83 (0.79–0.88)**	** < 0.001**
Study and life adaptation	924	**0.95 (0.91–0.99)**	**0.015**	992	**0.86 (0.82–0.91)**	** < 0.001**
School environment factors						
Teacher-student relationship						
Positive relationship	924	1.00 (0.98–1.02)	0.862	992	**0.98 (0.96–1.00)**	**0.065**
Conflict relationship	924	**1.11 (1.07–1.16)**	** < 0.001**	992	**1.10 (1.04–1.15)**	** < 0.001**
Peer relationship						
Social emotion	924	0.99 (0.96–1.02)	0.424	992	**0.96 (0.92–1.01)**	**0.086**
Interpersonal interaction	924	0.97 (0.94–1.01)	0.131	992	**0.91 (0.87–0.95)**	** < 0.001**
Interpersonal harmony	924	**0.97 (0.95–0.99)**	**0.017**	992	**0.93 (0.90–0.96)**	** < 0.001**
Family environment factors						
Expressiveness	924	**0.82 (0.70–0.90)**	** < 0.001**	992	**0.78 (0.71–0.86)**	** < 0.001**
Conflict	924	**1.28 (1.19–1.38)**	** < 0.001**	992	**1.26 (1.17–1.36)**	** < 0.001**
Independence	924	1.01 (0.92–1.11)	0.759	992	0.96 (0.86–1.07)	0.477
Achievement orientation	924	0.95 (0.87–1.04)	0.311	992	1.06 (0.96–1.17)	0.240
Intellectual-cultural orientation	924	**0.94 (0.87–1.01)**	**0.090**	992	**0.90 (0.83–0.97)**	**0.008**
Active-recreational orientation	924	**0.90 (0.84–0.96)**	**0.003**	992	0.96 (0.89–1.04)	0.315
Moral-religious emphasis	924	**0.85 (0.77–0.94)**	**0.001**	992	**0.91 (0.82–1.01)**	**0.078**
Organization	924	**0.76 (0.69–0.83)**	** < 0.001**	992	**0.76 (0.69–0.84)**	** < 0.001**
Control	924	1.06 (0.98–1.14)	0.147	992	0.99 (0.91–1.07)	0.768
Cohesion	924	**0.82 (0.76–0.89)**	** < 0.001**	992	**0.81 (0.75–0.87)**	** < 0.001**
*Psychological factors*						
Symptom Check List-90 scores						
Obsessive–compulsive	924	**1.09 (1.07–1.11**)	** < 0.001**	992	**1.14 (1.12–1.17)**	** < 0.001**
Somatic complaint	924	**1.12 (1.09–1.14**)	** < 0.001**	992	**1.13 (1.11–1.16**)	** < 0.001**
Interpersonal sensitivity	924	**1.11 (1.08–1.13**)	** < 0.001**	992	**1.16 (1.13–1.19)**	** < 0.001**
Depression	924	**1.08 (1.07–1.10**)	** < 0.001**	992	**1.11 (1.09–1.13)**	** < 0.001**
Anxiety	924	**1.13 (1.10–1.16**)	** < 0.001**	992	**1.16 (1.13–1.18)**	** < 0.001**
Hostility	924	**1.19 (1.15–1.23**)	** < 0.001**	992	**1.23 (1.19–1.28)**	** < 0.001**
Phobic anxiety	924	**1.16 (1.13–1.20**)	** < 0.001**	992	**1.18 (1.14–1.21)**	** < 0.001**
Paranoid ideation	924	**1.20 (1.16–1.24**)	** < 0.001**	992	**1.28 (1.23–1.33)**	** < 0.001**
Psychoticism	924	**1.13 (1.10–1.16**)	** < 0.001**	992	**1.17 (1.14–1.20)**	** < 0.001**
Diet/sleeping	924	**1.18 (1.14–1.21**)	** < 0.001**	992	**1.23 (1.19–1.27)**	** < 0.001**

*ADSs* Adolescent dissociative symptoms, OR Odds ratio, CI Confidence interval.

*Data in bold are statistically significant (*P* < 0.10).

To further explore the potential gender differences in the association between ADSs and impact factors, we performed gender-stratified, multivariate logistic regression analyses. Analyses showed that the conflict relationship of teacher-student [adjusted odds ratio (AOR) 1.06, 95% CI 1.01–1.10], family expressiveness (AOR 0.87, 95% CI 0.78–0.98), family conflict (AOR 1.15, 95% CI 1.05–1.27), family organization (AOR 0.88, 95% CI 0.78–0.99), and family cohesion (AOR 0.87, 95% CI 0.77–0.99) were linked to ADSs only in males, while somatic complaint (AOR 1.04, 95% CI 1.00–1.08) and paranoid ideation scores (AOR 1.09, 95% CI 1.01–1.19) were significantly associated with female ADSs only (Table 3).

**Table 3:** Multivariate logistic regression analyses were stratified for the association between impact factors and ADSs by gender during the coronavirus disease 2019 pandemic (*N* = 1,916).

Variables	Males (*n* = 924)*		*P-*value	Females (*n* = 992)*		*P*-value
	No	Adjusted OR (95% CI)		No	Adjusted OR (95% CI)	
**Sociodemographic characteristics**						
Age (years)	924	NA	NA	992	1.07 (0.94–1.21)	0.055
Residence area						
Rural	625	1.43 (1.00–2.08)	0.055	695	NA	NA
Urban	299	Reference		297	Reference	
**Environment factors**						
Social adjustment status						
Emotional adaptation	924	0.95 (0.90–1.02)	0.140	992	1.01 (0.92–1.10)	0.876
Social adaptation	924	1.00 (0.93–1.08)	0.987	992	0.92 (0.83–1.02)	1.00
Study and life adaptation	924	1.04 (0.97–1.12)	0.218	992	1.00 (0.92–1.09)	0.963
School environment factors						
Teacher-student relationship						
Positive relationship	924	NA	NA	992	1.01 (0.98–1.05)	0.383
Conflict relationship	924	**1.06 (1.01–1.10)**	**0.016**	992	1.00 (0.94–1.07)	0.933
Peer relationship						
Social emotion	924	NA	NA	992	1.07 (0.99–1.15)	0.077
Interpersonal interaction	924	NA	NA	992	0.94 (0.87–1.02)	0.134
Interpersonal harmony	924	1.02 (0.98–1.06)	0.278	992	1.01 (0.95–1.08)	0.782
Family environment factors						
Expressiveness	924	**0.87 (0.78–0.98)**	**0.020**	992	0.95 (0.83–1.09)	0.454
Conflict	924	**1.15 (1.05–1.27)**	**0.004**	992	1.01 (0.91–1.12)	0.852
Intellectual-cultural orientation	924	1.01 (0.91–1.12)	0.900	992	1.01 (0.90–1.13)	0.850
Active-recreational orientation	924	1.01 (0.91–1.11)	0.923	992	NA	NA
Moral-religious emphasis	924	0.96 (0.86–1.09)	0.549	992	1.00 (0.88–1.15)	0.967
Organization	924	**0.88 (0.78–0.99)**	**0.036**	992	0.93 (0.81–1.07)	0.312
Cohesion	924	**0.87 (0.77–0.99)**	**0.032**	992	1.04 (0.92–1.19)	0.517
**Psychological factors**						
Symptom Check List-90 scores						
Obsessive–compulsive	924	0.97 (0.93–1.02)	0.219	992	1.01 (0.96–1.06)	0.832
Somatic complaint	924	1.01 (0.97–1.06)	0.540	992	**1.04 (1.00–1.08**)	**0.041**
Interpersonal sensitivity	924	0.97 (0.91–1.03)	0.306	992	0.99 (0.93–1.05)	0.750
Depression	924	1.01 (0.97–1.06)	0.557	992	1.01 (0.97–1.06)	0.493
Anxiety	924	1.06 (0.99–1.14)	0.086	992	1.00 (0.94–1.07)	0.935
Hostility	924	1.02 (0.95–1.09)	0.608	992	1.04 (0.97–1.11)	0.310
Phobic anxiety	924	1.02 (0.96–1.09)	0.511	992	1.00 (0.95–1.06)	0.964
Paranoid ideation	924	1.08 (0.99–1.18)	0.086	992	**1.09 (1.01–1.19)**	**0.035**
Psychoticism	924	0.97 (0.91–1.04)	0.435	992	1.01 (0.95–1.08)	0.709
Diet/sleeping	924	1.05 (0.99–1.12)	0.126	992	1.02 (0.96–1.09)	0.538

*ADSs* Adolescent dissociative symptoms, *OR* Odds ratio, *CI* Confidence interval, *NA* Not available.

*Data in bold are statistically significant (*P* ≤ 0.05).

## Discussion

Unfortunately, there have been limited reports to date designed to evaluate the dissociative score, the prevalence and impact factors of ADSs during the COVID-19 pandemic. In the present study, we performed a two-center cross-sectional study with stratification by gender to explore dissociative scores, the prevalence, and impact factors of ADSs during the COVID-19 pandemic. Our results might provide more reliable evidence in developing prevention and intervention measures of ADSs and guiding mental health executives’ decision-making (e.g., formulate the effective surveillance, assessment and intervention programmes for ADSs). We anticipate that these findings will be useful in reducing the risk of ADSs and the design of clinical interventions for preventing ADSs. In addition, this study will provide a scientific basis for the gender-specific prevention and treatment of ADSs in pandemics.

### Gender differences in the prevalence of ADSs

To the best of the authors' knowledge, few similar studies have been previously conducted on a large scale of ADSs in China during the COVID-19 pandemic. Prior studies have noted that the intervention in risk factors was important from depression tendency to depression or other adverse outcomes^42,43^. However, very little was found in the literature on the question of ADSs intervention. The current study found that DSs seemed to be prevalent among adolescents during the COVID-19 pandemic. Compared with a prior study^44^, which reported a prevalence of 7% of post-traumatic stress symptoms a month after the COVID-19 epidemic. This DSs prevalence seems to have an even greater impact on adolescents. The levels observed in this investigation are also far beyond those observed by Kihlstrom et al. in the total population^45^. The yields in this investigation were also higher compared to our earlier similarity observations^34,46^.

Clearly, attention needs to be focused on the prevalence rate of ADSs, which is higher than expected. A possible explanation for this might be that the global pandemic of COVID-19 is creating distress and exacerbating psychological health problems in adolescents during this study period. Recent studies have revealed that trauma-related disorders (i.e., Post-traumatic stress disorder, autism spectrum disorder, and DD) were particularly prevalent during the COVID-19 pandemic^47,48^. Thus, the strong association between trauma-related symptoms (e.g., ADSs) and environmental factors was indirectly confirmed. The ADSs may be a serious public health problem in the future of China. According to these data, we can infer that an effective intervention strategy is urgently needed and further exploring the mechanism underlying is urgently required for reducing the prevalence of ADSs.

Another important finding was that the prevalence of ADSs was higher in males than in females. Our study has suggested that environmental factors, such as school factors (i.e., the conflict relationship of teacher-student) and family factors (i.e., family expressiveness, family conflict, family organization, and family cohesion), seemed to affect more male ADSs than female ADSs. Currently, COVID-19 has caused a global pandemic. The higher prevalence of males might be affected by the environmental factor of COVID-19 pandemic. Another reason may be that long-term infection control measures for COVID-19 seem to be easier to trigger TSRs among male adolescents^49^. This finding was contrary to previous similarity studies which have suggested that the prevalence of males was lower than females for depression tendencies, hysterical tendencies, and somatization tendencies^34,46,50^. In fact, as Rania et al*.*^51^ argued, males seemed to have worsened this dimension by perceiving more loneliness due to infection control measures during COVID-19. The present study raised the possibility that it would be beneficial to reduce the ADSs prevalence by using the gender-specific intervention.

This finding was unexpected and suggested that the prevalence of ADSs was higher in rural areas than in urban areas among males. However, this difference was not found in female ADSs. It is difficult to explain this result, but it might be related to psychological characteristics of male adolescents. Prior studies indicated that the mental health of male adolescents was more likely to be affected by environmental factors^46^. The superposition of poor environmental conditions and COVID-19 pandemic seems to aggravate the mental health problems of male adolescents in rural areas. This finding, while preliminary, suggests that there is an urgent need to reduce male ADSs in rural adolescents.

### Gender differences in the impact factors of ADSs

In our study, the conflict relationships of teacher-student, family expressiveness, family conflict, family organization, and family cohesion contributed to male ADSs only. Simultaneously, somatic complaint and paranoid ideation scores were only associated with female ADSs. These results provided further support for the hypothesis that environmental factors (i.e., school and family environments) seemed to affect more male ADSs than female ADSs; correspondingly, the impact on individual psychological factors seemed to be more female ADSs than male ADSs during the COVID-19 pandemic. It is difficult to explain these findings, but it might be related to the difference in personality traits between females and males^52^. Female adolescents may suffer more re-experiencing, negative alterations than males in cognitions and mood, and hyperarousal symptoms^53^. Findings indicated, as has also been observed in USA and China^54,55^, that women were more emotionally vulnerable to the effects of COVID-19 context than men. Within these personality traits, some studies suggested that male adolescents had higher levels of extraversion than females, while females presented higher values in conscientiousness and neuroticism^56,57^. Indeed, there was also a gender difference in stress-coping among adolescents, where females have shown greater stress and lower stress-coping abilities than males^57^, thus supporting our findings.

Prior studies suggested that male adolescents might be more psychologically mature compared with female adolescents in the same age category^58^. Male adolescents can get good mental health through their personality traits and mutually beneficial interpersonal relationships to reduce the impact on mental factors for the risk of ADSs^59^. In addition, female adolescents are relatively more vulnerable to changes in psychological quality than male adolescents, and they are more easily influenced by individual psychological factors^59^. Thus, girls with poor psychology maturity are easier to increase the risk of ADSs during COVID-19. Several reports have shown that the traits of masculinity or femininity could have affects beyond the psychological maturity of males and females^60,61^. For this reason, they produce perceived gender differences, which includes differences in terms of social relationships, personal resources, coping strategies, stressors, and vulnerabilities^60–62^. Evidence supported the psychological well-being benefited the mental development of females more than males^63^. These findings might help us provide gender-specific intervention strategies for ADSs. For instance, to decrease the risk of ADSs, interventions in environmental factors are urgently needed for male ADSs, and the interventions of mental health are urgently needed for female ADSs. Mental therapy, and the prevention interventions in family and school risk factors, are key to addressing adolescents at a high risk of ADSs.

Notably, our study suggested that family factors (such as expressiveness, conflict, organization, and cohesion) were key factors that affect the risk of male ADSs. This finding was consistent with that of Modestin et al.^64^ who indicated that environmental factors contributed to dissociative experiences in young non-patients. The data showed that home isolation had increased the perception of loneliness more in men than in women during the COVID-19 pandemic^51^. Considering the current epidemic situation, COVID-19 may further aggravate the loneliness of male adolescents. Prior studies have noted the importance of a positive family climate, which might provide safe surroundings for high-risk adolescents^34,64,65^. Stable family circumstances could promote positive mental health, optimize learning and living, and prevent health-related events^34,65^. Previous research has established that family conflict had unique and direct associations with adolescent mental health^66^. We compared our findings with previous conclusions^34,46,65^. On the one hand, these results seemed to be consistent with our earlier studies which found somatization tendencies were significantly associated with family conflict among adolescents^46^. On the other hand, interestingly, there were some notable differences that should be highlighted. For example, we found that family achievement orientation was significantly linked to hysterical tendencies only in male adolescents^34^. Whilst family cohesion was significantly associated with female hysterical tendencies only^34^. The present results were significant in at least two major respects. First, family interventions were key to reducing the risk of male ADSs. Second, education and mental health professionals should perform distinctive intervention and prevention for adolescent psychological problems during COVID-19.

Several lines of evidence suggested that psychological problems were associated with the course of paranoid ideation or somatic complaint in adolescents^67,68^. These findings further supported the idea of our study. In the present study, female ADSs were predicted by somatization and paranoia symptoms using a categorical regression analysis. This also matched the clinical observations of dissociative symptoms in adolescence reported by Mutluer et al*.*^69^. In summary, we should focus on the mental health of females in monitoring ADSs. Interestingly, the present data suggested that male adolescents had higher scores on ADSs during the COVID-19 pandemic than females. Contrary to the results of Zhang et al*.*^19^*,* where females showed higher levels of ADSs scores than males. However, these differences could be attributed to the difference in the sociocultural context (China–Europe), as well as the context of the COVID-19. These data should be brought to the attention of political decision-makers: we also need to focus on the general adolescent population during the COVID-19 pandemic.

Currently, we still do not fully available a standard approach to measuring ADSs at a fundamental level. The screening of ADSs is somewhat subjective. Thus, the reliability of studies depends mainly on the availability of good-quality data. In this study, we used a two-stage appraisal procedure to identify ADSs based on both subjective and objective evaluation methods; this reduced subjectivity in our screening of ADSs and correspondingly improved the internal validity of the study. Additionally, to enhance the response rate and minimize possible selection bias, we used a multiphase, stratified, cluster sampling method and randomly drew a large sample from a school-based population.

The multifactorial analysis of factors related to the ADSs risk during COVID-19 may be a useful tool to measure the associated TSRs among adolescents to explain and prevent the mental outcomes of COVID-19 pandemic. The use and assessment of questionnaires may provide significant information to prevent and control the mental disorders with COVID-19 pandemic in a short period. Knowledge of this related information could be used by various epidemic response institutions to perform multidisciplinary interventions to reduce this risk and, thus, adolescents' TSRs in the face of the COVID-19.

#### Limitations

Several limitations should be noted. First, we used the self-reported survey methods, which might decrease the validity of report because of reporting bias or recall bias. However, we used the confidential nature of the questionnaire to effectively reduce under-reporting in our study. Second, some outcomes might be misclassified due to no "gold standard" measurement method of ADSs. Thus, we might underestimate the associations between ADSs and impact factors during the COVID-19 pandemic. However, to reduce this underestimation, we used to integrate both subjective and objective methods to measure ADSs. Third, we only included on-campus adolescents; off-campus adolescents were not sampled in our studies. Thus, these findings may not be extrapolated to all adolescent populations. Notwithstanding these limitations, the study suggests that early intervention with prevention is urgently needed for adolescent psychological problems during COVID-19.

## Conclusions

This study has identified that DSs seem to be prevalent (23.12%) in adolescents across Hangzhou City studied during the COVID-19 pandemic. The prevalence of male ADSs was significantly higher compared with female ADSs. The research has also shown that gender differences in terms of the impact factors of ADSs are very significant. The environmental factors (i.e., school and family environments) seem to affect more male ADSs than female ADSs; correspondingly, the impact on individual psychological factors (i.e., paranoid ideation and somatic complaints) seems to be more female ADSs than male ADSs during the COVID-19 pandemic.

Overall, the present study assessed the gender characteristics of ADSs during the COVID-19 pandemic based on dissociative scores, the prevalence, and the impact factors of social-demographic, environment, and psychology. These findings provided a scientific foundation for the sex-specific prevention and intervention of ADSs in pandemics. This study strengthens the idea that we urgently need gender-specific prevention and intervention to reduce the prevalence and risk of ADSs, preventing further ADD or other adverse outcomes during COVID-19. To reduce the risk of male ADSs, we should focus on the prevention and intervention of environmental factors (such as teacher-student relationships and family factors). To decrease the risk of female ADSs, we should promote the mental well-being of female adolescents through more effective mental health promotion intervention programs. The present study has been one of the first attempts to scrutinize the prevalence and impact factors of ADSs during the COVID-19 pandemic. This will lay the groundwork for future research. Despite its exploratory nature, this study offers some insight into early prevention and intervention of ADD. Finally, we need to use large-scale experimental research to identify how to improve adolescents' psychological quality and change environmental risk factors. In addition, further research needs to examine more closely the links between ADSs and ADD or other adverse outcomes.

## Data Availability

The datasets generated and/or analysed during the current study are not publicly available due to confidentiality issues, but are available from the corresponding author on reasonable request. Q.L.C (chenghzcdc@sina.com) should be contacted if someone wants to request the data from this study.

## Abbreviations

COVID-19: Coronavirus disease 2019
TSRs: Traumatic stress reactions
ADD: Adolescent dissociative disorder
DSs: Dissociative symptoms
DSM-5: Diagnostic and statistical manual of mental disorders 5th edition
ADSs: Adolescent dissociative symptoms
DD: Dissociative disorder
CI: Confidence intervals
SHSS: School health surveillance system
A-DES: Adolescent dissociative experience scale
SCID-D: Structured clinical interview for DSM-5- dissociative disorders
CASA: Chinese adaptation scale for adolescents
STRS: Student–teacher relationship scale
STRS-CV: Student–teacher relationship scale-Chinese version
CPRI: Chinese version of the peer relationship inventory
FES-CV: Chinese version of family environment scale
SCL-90-CV: Chinese version of symptom checklist-90
MCMC: Markov chain monte carlo
MI: Multiple imputation
SD: Standard deviation
AOR: Adjusted odds ratio

## References

[1] Reynolds LM (2019). Early adolescence is a critical period for the maturation of inhibitory behavior. Cereb. Cortex.

[2] De France K, Hollenstein T (2021). Implicit theories of emotion and mental health during adolescence: The mediating role of emotion regulation. Cogn. Emot..

[3] De France K, Hancock GR, Stack DM, Serbin LA, Hollenstein T (2022). The mental health implications of COVID-19 for adolescents: Follow-up of a four-wave longitudinal study during the pandemic. Am. Psychol..

[4] Hu Y, Qian Y (2021). COVID-19 and adolescent mental health in the United Kingdom. J. Adolesc. Health.

[5] Duan L (2020). An investigation of mental health status of children and adolescents in china during the outbreak of COVID-19. J. Affect. Disord.

[6] Kilincel S, Kilincel O, Muratdagi G, Aydin A, Usta MB (2021). Factors affecting the anxiety levels of adolescents in home-quarantine during COVID-19 pandemic in Turkey. Asia Pac. Psychiatry.

[7] Oosterhoff B, Palmer CA (2020). Attitudes and psychological factors associated with news monitoring, social distancing, disinfecting, and hoarding behaviors among US adolescents during the coronavirus disease 2019 pandemic. JAMA Pediatr..

[8] Xu H (2021). Increased symptoms of post-traumatic stress in school students soon after the start of the COVID-19 outbreak in China. BMC Psychiatry.

[9] Ferrante G (2022). The emotional side of post-traumatic stress reaction during COVID-19 pandemic: An Italian survey. BMC Public Health.

[10] Silberg JL, Lewis M, Rudolph K (2014). Dissociative Disorders in Children and Adolescents. Handbook of Developmental Psychopathology.

[11] Ross J, Armour C, Kerig PK, Kidwell MC, Kilshaw RE (2020). A network analysis of posttraumatic stress disorder and dissociation in trauma-exposed adolescents. J. Anxiety Disord..

[12] Zoroglu SS (2003). Suicide attempt and self-mutilation among Turkish high school students in relation with abuse, neglect and dissociation. Psychiatry Clin.l Neurosci..

[13] Goffinet SJL, Beine A (2018). Prevalence of dissociative symptoms in adolescent psychiatric inpatients. Eur. J. Trauma Dissociation.

[14] Sar V, Onder C, Kilincaslan A, Zoroglu SS, Alyanak B (2014). Dissociative identity disorder among adolescents: Prevalence in a university psychiatric outpatient unit. J. Trauma Dissociation.

[15] Luoni C, Agosti M, Crugnola S, Rossi G, Termine C (2018). Psychopathology, dissociation and somatic symptoms in adolescents who were exposed to traumatic experiences. Front. Psychol..

[16] Lo Iacono L, Carola V (2018). The impact of adolescent stress experiences on neurobiological development. Semin. Cell Dev. Biol..

[17] Bolinski RS (2022). The impact of the COVID-19 pandemic on drug use behaviors, fentanyl exposure, and harm reduction service support among people who use drugs in rural settings. Int. J. Environ. Res. Public Health.

[18] Reinhardt M (2022). Dissociative tendencies aggregate the impact of negative life events on non-suicidal self-injury among male juvenile delinquents. Arch. Suicide Res..

[19] Zhang X, Gatzke-Kopp LM (2022). Exposure to parental aggression and the development of psychopathology in young children: The mediating role of early dissociative symptoms. J. Interpers. Violence.

[20] Noël X, Saeremans M, Kornreich C, Jaafari N (2018). Dissociative tendencies and alcohol use disorder. Curr. Addict. Rep..

[21] Lyssenko L (2018). Dissociation in psychiatric disorders: A meta-analysis of studies using the dissociative experiences scale. Am. J. Psychiatry.

[22] Hoyos C (2019). The role of dissociation and abuse among adolescents who self-harm. Aust. N. Z. J. Psychiatry.

[23] Kefeli MC, Turow RG, Yildirim A, Boysan M (2018). Childhood maltreatment is associated with attachment insecurities, dissociation and alexithymia in bipolar disorder. Psychiatry Res..

[24] Thompson A (2016). Do affective or dissociative symptoms mediate the association between childhood sexual trauma and transition to psychosis in an ultra-high risk cohort?. Psychiatry Res..

[25] Fung HW, Ross CA, Yu CK, Lau EK (2019). Adverse childhood experiences and dissociation among Hong Kong mental health service users. J. Trauma Dissociation.

[26] Fung HW, Chan C, Ross CA, Wang EKS (2021). Clinical features of a Chinese sample with self-reported symptoms of pathological dissociation. J. Trauma Dissociation.

[27] Ford JD, Charak R, Modrowski CA, Kerig PK (2018). PTSD and dissociation symptoms as mediators of the relationship between polyvictimization and psychosocial and behavioral problems among justice-involved adolescents. J. Trauma Dissociation.

[28] Vine V, Victor SE, Mohr H, Byrd AL, Stepp SD (2020). Adolescent suicide risk and experiences of dissociation in daily life. Psychiatry Res..

[29] Yamasaki S (2016). Dissociation mediates the relationship between peer victimization and hallucinatory experiences among early adolescents. Schizophr. Res. Cognit..

[30] Rodriguez Del Aguila M, Gonzalez-Ramirez A (2014). Sample size calculation. Allergol. Immunopathol..

[31] Putnam FW (1997). Dissociation in children and adolescents: A developmental perspective.

[32] Kisiel CL, Lyons JS (2002). Dissociation as a mediator of psychopathology among sexually abused children and adolescents. Am. J. Psychiatry.

[33] Kuroda N (2019). Low body mass index and low intelligence quotient are infection risk factors in vagus nerve stimulation. World Neurosurg..

[34] Cheng Q (2018). Gender differences in the prevalence and impact factors of hysterical tendencies in adolescents from three eastern Chinese provinces. Environ. Health Prev. Med..

[35] Zhang YB (2008). The current situation and relationship of self-cognition, self-evaluation and adaptation of Chinese junior high school students.

[36] Pianta RC (2001). The student-teacher relationship scale.

[37] Wei YH (1997). A study on structure model and factors influencing the self-esteem development in children and adolescents.

[38] Phillips MR, West CL, Shen Q, Zheng Y (1998). Comparison of schizophrenic patients' families and normal families in China, using Chinese versions of FACES-II and the family environment scales. Fam. Process.

[39] Zhou J, Yu J, Zhou Y, Qiu J (2021). Study of item text in the Chinese symptom checklist-90. Medicine.

[40] Zhao M, Feng Z, Yang G (2019). Improvement of mental health among Chinese plateau military personnel, 1993–2017: A cross-temporal meta-analysis of the symptom checklist-90. Neuropsychiatr. Dis. Treat..

[41] Mulla ZD, Seo B, Kalamegham R, Nuwayhid BS (2009). Multiple imputation for missing laboratory data: An example from infectious disease epidemiology. Ann. Epidemiol..

[42] Takahashi Y, Tamakoshi K (2014). Factors associated with early postpartum maternity blues and depression tendency among Japanese mothers with full-term healthy infants. Nagoya J. Med. Sci..

[43] Godha D, Shi L, Mavronicolas H (2010). Association between tendency towards depression and severity of rheumatoid arthritis from a national representative sample: The medical expenditure panel survey. Curr. Med. Res. Opin..

[44] Liu N (2020). Prevalence and predictors of PTSS during COVID-19 outbreak in China hardest-hit areas: Gender differences matter. Psychiatry Res..

[45] Kihlstrom JF, Glisky ML, Angiulo MJ (1994). Dissociative tendencies and dissociative disorders. J. Abnorm. Psychol..

[46] Cheng Q, Xu Y, Xie L, Hu Y, Lv Y (2019). Prevalence and environmental impact factors of somatization tendencies in eastern Chinese adolescents: A multicenter observational study. Cad. Saude Publica.

[47] Qi J, Sun R, Zhou X (2021). Network analysis of comorbid posttraumatic stress disorder and depression in adolescents across COVID-19 epidemic and Typhoon Lekima. J. Affect. Disord..

[48] Alhuzimi T (2021). Stress and emotional wellbeing of parents due to change in routine for children with autism spectrum disorder (ASD) at home during COVID-19 pandemic in Saudi Arabia. Res. Dev. Disabil..

[49] Clemens V (2020). Potential effects of "social" distancing measures and school lockdown on child and adolescent mental health. Eur. Child Adolesc. Psychiatry.

[50] Suzuki H (2019). Socioeconomic and lifestyle factors associated with depressive tendencies in general Japanese men and women: Nippon Data 2010. Environ. Health Prev. Med..

[51] Rania N, Coppola I (2021). Psychological Impact of the lockdown in Italy due to the COVID-19 outbreak: Are there gender differences?. Front. Psychol..

[52] Gao W, Ping S, Liu X (2020). Gender differences in depression, anxiety, and stress among college students: A longitudinal study from China. J. Affect. Disord..

[53] Villalta L (2020). Complex post-traumatic stress symptoms in female adolescents: The role of emotion dysregulation in impairment and trauma exposure after an acute sexual assault. Eur. J. Psychotraumatol..

[54] Hawes MT, Szenczy AK, Klein DN, Hajcak G, Nelson BD (2021). Increases in depression and anxiety symptoms in adolescents and young adults during the COVID-19 pandemic. Psychol. Med..

[55] Chen X (2021). Depression, anxiety and associated factors among Chinese adolescents during the COVID-19 outbreak: A comparison of two cross-sectional studies. Transl. Psychiatry.

[56] Evans BE (2016). Neuroticism and extraversion in relation to physiological stress reactivity during adolescence. Biol. Psychol..

[57] Rodriguez-Besteiro S, Tornero-Aguilera JF, Fernandez-Lucas J, Clemente-Suarez VJ (2021). Gender differences in the COVID-19 pandemic risk perception, psychology, and behaviors of Spanish university students. Int. J. Environ. Res. Public Health..

[58] Liang L, Zhou D, Yuan C, Shao A, Bian Y (2016). Gender differences in the relationship between internet addiction and depression: A cross-lagged study in Chinese adolescents. Comput. Hum. Behav..

[59] Ethier KA (2006). Self-esteem, emotional distress and sexual behavior among adolescent females: Inter-relationships and temporal effects. J. Adolesc. Health.

[60] Rosenfield S, Mouzon D (2013). Gender and mental health Handbook of the sociology of mental health.

[61] Allen MT, Bocek CM, Burch AE (2011). Gender differences and the relationships of perceived background stress and psychological distress with cardiovascular responses to laboratory stressors. Int. J. Psychophysiol..

[62] Crocetti E (2019). Developing morality, competence, and sociability in adolescence: A longitudinal study of gender differences. J. Youth Adolesc..

[63] Matud MP, Lopez-Curbelo M, Fortes D (2019). Gender and psychological well-being. Int. J. Environ. Res. Public Health..

[64] Modestin J, Lötscher K, Erni T (2002). Dissociative experiences and their correlates in young non-patients. Psychol. Psychother..

[65] Zhao G, Xie L, Xu Y, Cheng Q (2018). A multicenter cross-sectional study on the prevalence and impact factors of hysteria tendency in the Eastern Chinese adolescents. Iran. J. Public Health.

[66] Kelly AB (2016). Depressed mood during early to middle adolescence: A Bi-national longitudinal study of the unique impact of family conflict. J. Youth Adolesc..

[67] Saarinen A (2018). The co-occurrence between depressive symptoms and paranoid ideation: A population-based longitudinal study. J. Affect. Disord..

[68] Kim JHJ (2019). Cultural variation in temporal associations among somatic complaints, anxiety, and depressive symptoms in adolescence. J. Psychosom. Res..

[69] Mutluer T (2021). Psychopathology and dissociation among boarding school students in Eastern Turkey. J. Child Adolesc. Trauma.

